# PERCEPTION OF HARASSMENT AMONG FEMALE SURGEONS

**DOI:** 10.1590/0100-6991e-20213123

**Published:** 2021-08-13

**Authors:** ELIZABETH G SANTOS, LIA ROQUE, MARIA CRISTINA MAYA, RENI CECILIA MOREIRA, FERNANDA LAGE LIMA, M ISABEL T. D. CORREIA

**Affiliations:** 1 - Hospital Universitário Clementino Fraga Filho - UFRJ, Member Of The Women Surgeons Committee - MD, PhD, ECBC, FACS - Rio de Janeiro - RJ - Brasil; 2 - Universidade do Estado do Rio de Janeiro, Member Of The Women Surgeons Committee - MD, PhD, TCBC - Rio de Janeiro - RJ - Brasil; 3 - Universidade Federal do Rio de Janeiro, Member Of The Women Surgeons Committee - TCBC, PhD - Rio de Janeiro - RJ - Brasil; 4 - UNIBH, Member Of The Women Surgeons Committee - MD, TCBC, FACS, TSBCO, TSBC - Belo Horizonte - MG - Brasil; 5 - Universidade Federal do Acre, Secretaria de Estado de Saúde do Acre, Member Of The Women Surgeons Committee - MD, MsC, TCBC, FACS - Rio Branco - AC - Brasil; 6 - Universidade Federal de Minas Gerais, Member of ETERNA, Rede Mater Dei, Member Of The Women Surgeons Committee - MD, PhD, TCBC, FACS, FASPEN - Belo Horizonte - MG - Brasil

**Keywords:** Surgery, Women, Gender Identity, Prejudice, Cirurgia, Mulheres, Identidade de Gênero, Preconceito

## Abstract

**Introduction:**

the attraction of women by Surgery has always existed. Although Surgery has been considered a specialty for men, several women chose it, despite gender bias issues that have persisted over many years. Several obstacles have impacted the practice of women surgeons, leading them to abandon the profession, while others, perhaps bearers of a stronger spirit, managed to overcome them, and won.

**Objective:**

to assess the rates of perception of harassment against female surgeons as a cause of difficulty and negative feelings related to the specialty.

**Methods:**

we conducted a quantitative and qualitative (personal accounts) research through a questionnaire via Google Forms® sent to all women surgeons registered in the Brazilian College of Surgeons and in a WhatsApp women surgeons’ groups. The qualitative analysis was made with the Wordle® app.

**Results:**

from 821 questionnaires sent, we obtained 232 responses (28.2%). Harassment perception during training was 49.1% (n=114). From the women surgeons who perceived harassment, 56.1% reported having undergone different training than expected, with statistical significance (p<0.001). The question of having been treated differently due to being a woman also had an impact on harassment perception (77.2% harassed vs 47.5%; p<0.001). Physical (42.1% vs 6.8%) and emotional (92.1% vs 39.8%) threats were also different between groups.

**Conclusion:**

women surgeons still report great harassment perception, both moral and sexual, which impacts their feelings about the specialty.

## RESUMO

**Introdução:**

a atração das mulheres pela Cirurgia sempre existiu. Embora a Cirurgia seja considerada especialidade para homens, várias mulheres a escolheram, apesar de questões de preconceito de gênero que têm se mantido ao longo de vários anos. Vários obstáculos têm impactado na prática das cirurgiãs, levando-as a abandonar a profissão, mas outras, talvez, as de espírito mais forte, conseguiram superá-los e venceram.

**Objetivo:**

avaliar a taxas de percepção de assédio contra cirurgiãs como causa de dificuldade e sentimentos negativos relacionados com a especialidade.

**Método:**

pesquisa, quantitativa e qualitativa (relatos pessoais), realizada por meio de questionário via Google Forms® enviado para todas as cirurgiãs registradas no Colégio Brasileiro de Cirurgiões e em grupo WhatsApp de cirurgiãs. A análise qualitativa foi feita com o aplicativo Wordle®.

**Resultados:**

Foram enviados 821 questionários e obtidas 232 respostas (28,2%). A percepção de assédio durante o treinamento foi de 49.1% (n=114). As cirurgiãs com percepção de assédio foram estatisticamente aquelas que reportaram ter tido treinamento diferente do que ansiavam (56,1%) (p<0,001). O quesito tratamento distinto por ser mulher também impactou na percepção do assédio (77,2% assediadas vs 47,5%; p<0,001). Ameaça física (42,1% vs 6,8%) e emocional (92,1% vs 39,8%) também foram distintas entre os grupos.

**Conclusão:**

cirurgiãs ainda reportam grande percepção de assédio moral e sexual, o que impacta na forma de encarar a profissão.

## INTRODUCTION

Women have participated in Medicine since ancient times. The attraction to Surgery has always existed. The most notorious case is perhaps that of Margareth Ann Bulky, known as James Barry, the leading surgeon in the British Army for 40 years. Bulky transformed her appearance into a male and earned a medical degree from the Edinburgh University School of Medicine. She lived her entire public and private life as a man. She was recognized as having “great surgical skill, aggressive manners, and perfect aim”. Only after her death was it known that she was a woman. Such knowledge caused violent impact, but the scandal was hushed up and she was buried as she lived: James Barry^1^ ^,^ ^2^. 

The formation of a patriarchal society in which men were the providers and women the caregivers for a long time smothered the desire of women to enter Medicine and Surgery. Except for some periods and places, such as Old Egypt and Greece, where those who had the gift of healing were revered, women have always been seen as fragile elements that should be protected and restricted to the house. They have suffered prejudice, insults, and belittling. However, some have marked their presence, taking advantage of gaps found in different historical moments^3^. 

The Middle Ages represented a dark period for Medicine in general, especially for women, who were always under a male power: father, guardian, brother, or husband. Control over the feminine element came from the figure of Eve, who fed a misogynist feeling, rendering the woman to be more prone to sin by nature. The woman was seen as a threat and, therefore, should be subjected to male control. Not coincidentally, many were accused and sent to the fire for witchcraft and for possessing knowledge of the art of healing. These were mostly single, widows, or women who lived separated from their husbands and children^4^. 

Studies show that gender bias in Medicine is a recurring problem in many countries, even in the Western world, especially in the General Surgery Specialty. Bruce et. al., in 2015, showed that 88% of women perceived prejudice during training5. Even today, it is common for almost all of those who chose this specialty to have to face prejudices and commonly hear throughout their professional lives that “women cannot/should not be surgeons”^5^. 

Prejudice against women who wanted to be surgeons has grown on an exponential scale over the years. With so many obstacles, many give up the dream, but others, perhaps with stronger spirit, manage to overcome them. Still, even those who are in leadership positions many times only passed from a glass ceiling to a glass cliff status^6^. Therefore, the aim of this study was to assess the rates of harassment perception among women surgeons and how this could influence their training and feelings about the profession throughout their career. 

## METHODS

This is a cross-sectional study carried out by a semi-structured questionnaire (Appendix 1) built on Google Forms®. We sent it to all female surgeons registered in the Brazilian College of Surgeons and to those belonging to the “Women in Surgery” WhatsApp group. We surveyed both residents and staff surgeons, in three phases separated by 30 day intervals, between April and November 2020. We sent 821 questionnaires. At the end of the questionnaire, there was the possibility of inserting comments on what to say to a woman intern who is thinking about pursuing General Surgery as a specialty (qualitative analysis). We configured the system not to collect email addresses, thus not allowing for respondent identification. Therefore, the free and informed consent term was waived, as determined by RESOLUTION No. 510, OF APRIL 7, 2016. However, by accessing the link, the respondent implicitly granted authorization for the use of the data obtained. 

We used the chi square test to assess differences between groups, with values of p<0.05 considered statistically significant. We subjected harassment association measures to univariate logistic regression (RL) analysis, including binary and ordinal variables, in which the dependent variable was perceived harassment. We considered a 95% confidence interval (CI), and we used the version 10.1 of the statistical software STATA (College Station, USA) for all analyzes. 

We carried out the qualitative analysis with a word cloud built with the answers to the open question. We used the heuristic analysis method, whose purpose is to outline paths for what to observe in a group of texts. We used Wordle® to make the word cloud.

## RESULTS

We received 234 responses (28.2%). The prevalence of reported harassment perception during training or professional life was 49.1% (n=114). Table 1 has the record of responses between the groups of positive and negative harassment perception. 

**Table 1:** Frequency of structured questions’ responses.

	Perception of Harassment		
Question	Yes (%)	No (%)	p-value
Expected training	56.1	85.6	<0.001
Adequate number of operated patients	64.0	68.6	0.458
Specific preceptor designated	33.3	33.0	0.964
Medical staff surgeons with < 10 years of training	79.8	70.3	0.095
Presence of staff surgeons of both sexes	73.6	74.6	0.877
Staff surgeons close to the resident	82.4	89.6	0.184
Surgeon sex makes a difference in training	17.5	8.5	0.04
Woman resident treated differently	77.2	47.5	<0.001
Female resident treated differently by male residents	66.7	36.4	<0.001
Female resident treated differently by staff surgeons	79.8	47.5	<0.001
Physical threat	42.1	6.8	<0.001
Emotional threat	92.1	39.8	<0.001
Fewer options due to being a female resident	49.1	16.9	<0.001
Negative impact on doctor-patient relationship for being a woman	19, 3	23.7	0.412
Doubt about reaching the end of training	53.5	27.1	<0.001
Choice for Surgery again	76.3	87.3	0.03
Glad to have chosen Surgery	86.8	89.8	0.478
Gratification greater than sadness	83.3	94.1	0.01
Harassment perception improved over time	59.6	36.4	<0.001

Women surgeons with positive harassment perception reported having received a worse surgical training than they expected during residency, with statistical significance (56.1% positive harassment perception vs. 85.6%, p<0.001). Similarly, these women also believed that occurred because they are women (77.2% vs. 47.5% without harassment perception, p<0.001). Such perception happened specifically in the treatment by male fellow residents and by all other staff surgeons, although the preceptors’ sex differed in the treatment of this specific group (17.5% vs 8.5% p=0.04). 

Women with perceived harassment reported a higher number of physical and emotional threats, as well as fewer surgical opportunities due to being female residents. Harassment perception caused doubt about reaching the end of training and about ever choosing General Surgery again (53.5% vs 27.1%). Surgeons with harassment perception reported a lower gratification/sadness ratio than the non-harassed (83.3% vs. 94.1%). Finally, these surgeons had the impression that the harassment perception improved over time, ie, the initial interpretation of what would be harassment was modified by professional experience (59.6% vs 36.4%). 

Residents with harassment perception were 78.5% less likely to have had adequate training and felt treated differently by staff surgeons 4.38 times more (Table 2). Similarly, they were 10 times more exposed to physical violence and 17.62 times more to emotional violence (p<0.001). During training, harassment perception translated in 4.74 times more difficulty in having surgical opportunities. Perceived harassment decreased by 54.0% the chances to opt for residence in Surgery again. Moreover, it had a negative impact on the sense of gratification being greater than sadness (68.0%) by having chosen to be a surgeon. These surgeons were also more likely (2.57 times) to perceive that harassment improved throughout their surgical experience.

**Table 2:** Univariate analysis between harassment perception associations. .

Question	Odds Ratio	95% CI	p-value
Expected training	0.21	0.11-0.41	<0.001
Adequate number of operated patients	0.81	0.47-1.40	0.458
Specific preceptor designated	1.01	0.58-1.75	0.965
Medical staff surgeons with < 10 years of training	1.66	0.91-3.05	0.097
Presence of staff surgeons of both sexes	0.95	0.53-1.71	0.877
Staff surgeons close to the resident	0.54	0.21-1.35	0.189
Surgeon sex makes a difference in training	2.29	1.02-5.15	0 0 44
Woman resident treated differently	3.74	2.12-6.61	<0.001
Female resident treated differently by male residents	3.48	2.03-5.98	<0.001
Female resident treated differently by staff surgeons	4.38	2.44-7.85	<0.001
Physical threat	10	4.46-22.44	<0.001
Emotional threat	17.62	8.13-38.22	<0.001
Fewer options for being a female resident	4.73	2.58-8.66	<0.001
Negative impact on doctor-patient relationship for being a woman	0.96	0.74-1.26	0.814
Doubt about reaching the end of training	3.09	1.78-5.35	<0.001
Choice for Surgery again	0.46	0.23-0.94	0.032
Glad to have chosen Surgery	0.74	0.33-1.67	0.48
Gratification greater than sadness	0.32	0.13-0.78	0.013
Harassment perception improved over time	2.57	1.52-4.38	<0.001

The qualitative analysis with the Wordle® word cloud generated a list of the most used words women surgeons would recommend when talking to a young intern who would like to pursue with Surgery as a specialty. The application assigns weights to the words according to the number of times they are repeated and show them in different color and font size. The final picture is the expression of the messages^7^.

## DISCUSSION

Surgery, a predominantly considered male specialty, has been over many centuries chosen by several women who have faced numerous challenges. Among these, harassment perception still seems to have a great impact on these professionals’ lives. In the present study, we could confirm that harassment perception is still high among women (49.1%). Although there are currently more female medical students both in Brazil and in the world, the female workforce in surgical specialties, especially in General Surgery, remains exceptionally low^8^. This may be related to the harassment perception and to the difficulties they face, like the ones reported by our respondents. 

In recent years, several studies on the disparity and inequality between sexes have been published in the field of Surgery^9^ ^-^ ^12^. Some show alarming data concluding that women surgeons who suffer harassment are more prone to commit suicide and have burnout^13^. In these studies, female sex is associated with a lower number of performed operations and little self-confidence^13^. We found similar results, since women who had harassment perception indicated that they had fewer surgical opportunities and, in turn, would contraindicate the profession for younger women. 

**Chart 1:**
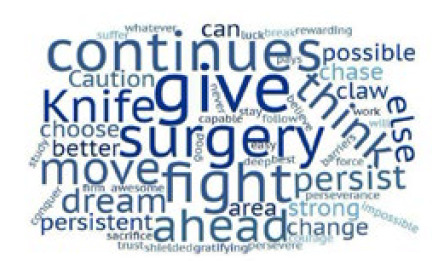
Word cloud representation of the free question texts.

Women have been discouraged to study Medicine for many years, and those who did, used various stratagems to practice Surgery. Some disguised as men, remaining hidden as “the male doctor” who took the laurels^14^. Others have changed their country, but between being burned alive and receiving glories, such as Prof. Angelita Habr-Gama, a long time has gone by^3^. It seems absurd, even surreal, that there is still a need to discuss harassment perception by female surgeons. Unfortunately, however, the world of Surgery is still extremely masculine, such as a Gentlemen’s Club, and women have to face many difficulties to stand out and be respected^9^ ^,^ ^11^ ^,^ ^12^. 

In several institutions, women surgeons are still seen as “troublemakers, problematic, and difficult to deal with”. They may have premenstrual syndrome and become pregnant^15^. It is still common for one to hear: “Can a woman practice Medicine?” or “Are women intelligent enough to be doctors”. It is true that such harassment is more common away from the big centers, but there are still entrance exams for Surgery residency programs in which the interviewer shows condescension when the candidate is female^16^. 

Some researchers have shown that men and women are seen differently by their evaluators. While men are described as having natural talent and being naturally prone to success, female success is seen as the result of hard work and only with the potential for success^5^ ^,^ ^17^ ^,^ ^18^. Hoops et al.^17^ identified a disparity in autonomy among General Surgery residents, with a significant reduction in autonomy among female residents. Gerull et al.^18^ state that disparities between men and women are particularly important in the field of Surgery. Bruce et al.^5^, in 2015, reported that 88% of women perceived gender discrimination during training, corroborating the findings of our study, in which 49.2% of respondents considered their training to be/having been worse (fewer surgical opportunities) for being a woman. 

Although Surgery has undergone many changes over the past two centuries, several people, among these patients and colleagues, when thinking of a surgeon, picture a smart and confident man^19^. For women, the path is always arduous, full of opposition, lack of support, including from family members, and with all kinds of prejudice. Harassment ranges from: “you will ruin your life as a woman if you choose a surgical career” to something like what Lucas-Championnière said: “Women cannot seriously pursue a medical career unless they are no longer women. Due to physiological laws, medical women are ambiguous, hermaphroditic, or asexual, monsters from all points of view”^20^. 

Many women later in their careers abandon the specialty after facing many difficulties and barriers to career advancement. Friction with colleagues, difficulty in assuming leadership positions, shifts and workload, as well as lack of support are the main reasons associated with this reality^5^. In our study, women who felt treated unequally (62.1%) were the ones that perceived harassment and said they would not pursue Surgery as a specialty again if they were aware of the barriers. Most said that the difference in treatment occurred by their resident colleagues and 64.4% said that staff surgeons treated them differently than the male colleagues. Perhaps for this reason, 26.3% of respondents reported a negative impact on the relationship with patients of both sexes due to being a woman. It is interesting to note that 40.1% of the professionals in our study responded that at some point they thought they would not be able to reach the end of training. Despite this negative side, the answers to the two last study questions were surprising to the extent that 81.9% of women said they would choose Surgery as a specialty again and, despite the difficulties, 88.4% are happy to be a surgeon. For these women, being a surgeon is more gratifying than saddening, even though 47.8% stated that harassment perception has not changed over time. 

We believe that the examples of female role models can Influence training women surgeons to develop resilience, pursue their careers, and not feel harmed. The Committee of Women Surgeons of the Brazilian College of Surgeons was created with this aim: to discuss the subject and to empower young female surgeons. We hope to inspire future generations so that they do not let themselves down and give up on the specialty they dreamed of. On the contrary, we want them to win and dedicate themselves to Surgery with determination and soul. 

Women still represent the minority in surgical specialties and this work can be an incentive for future gender equity in Surgery. However, there are limitations to be highlighted. We used questionnaires sent by the internet, which has been of great assistance in the current world. However, some authors indicate that data surveys with recruitment through the internet tend to have lower participation rates than traditional ones^21^ ^,^ ^22^. Usually, the response index is between 15% and 17%^23^, even in countries where Internet access is bigger than 86.0%. We obtained 232 responses (28.2%), which could be considered representative. However, not all surgeons in the country are members of the CBC or belong to the WhatsApp group to which the questionnaire was sent, so our conclusions may not reflect the feelings of other colleagues.

## CONCLUSION

Harassment perception is still highly prevalent among women surgeons, and this is a factor that interferes with training during residency and future decision-making, such as remaining in the specialty. 
